# Supplementary material for: Multiple Occurrences of a 168-Nucleotide Deletion in SARS-CoV-2 ORF8, Unnoticed by Standard Amplicon Sequencing and Variant Calling Pipelines
Source: Viruses. 2021 Sep 18;13(9):1870. doi: 10.3390/v13091870 (PMC8518987; doi:10.3390/v13091870)
Supplement: Supplementary file 1 [file viruses-13-01870-s001.zip › Brandt_et_al._Supplementary_Text_Viruses.pdf]

# Supplementary Text

## Detailed description of ARTIC fieldbioinformatics medaka-variant pipeline issues leading to undetected large deletions

In addition to the described problems with the ARTIC V3 primer set, we identified three analysis steps in the ARTIC pipeline (v1.2.1) as problematic with respect to identifying long deletions in the SARS-CoV-2 genome in general: (1) According to the recommendations of the ARTIC consortium (https://artic.network/ncov-2019/ncov2019-bioinformatics-sop.html), the lower boundary of the read length exclusion filter should be set to the length of the shortest amplicon in the utilized primer set. (2) The default value of 20 is used for the max_cigar_length option of longshot, which defines an upper boundary for consecutive indel characters (I/D/N) during allelotyping. (3) Longshot has a hardcoded upper boundary for the length of variants that are passed to longshot as potential variants for filtering. Any potential variant that is longer than 50 nt is ignored. Point (1) affects both versions of the ARTIC pipeline and leads to an apparent but inaccurate loss of coverage of any amplicon that contains a long deletion mutation, potentially excluding all associated reads from the analysis. Points (2) and (3) are specific to the Medaka version of the pipeline, potentially masking all long (>20 nt) deletions detected by medaka-variant with the reference sequence since they do not pass the longshot filter. Although we were able to call the Δ168 deletion using the ARTIC nanopolish pipeline, the detection would have been difficult with both versions of the pipeline due to problem (1), if the lower read length exclusion boundary would not have been reduced to 250 nt contrary to the ARTIC consortium's recommendations. contrary to the ARTIC consortium's recommendations.

Therefore, as we routinely used the medaka version of the ARTIC pipeline, which is also advised by the COG-UK consortium, we modified and recompiled longshot to prevent it from excluding long indels from the analysis as described in the supplementary material. Running the medaka version of the ARTIC pipeline with this modified longshot version and a "max_cigar_indel" setting of 250, we were able to call the deletion correctly in 43 out of 44 analyzed samples. All other variant calls were unaffected by these changes. We also reanalyzed our samples using nanopolish for variant calling, which resulted in correct deletion calling in 41 out of 44 samples. In the four unsuccessful cases, nanopolish called no variants at all, probably due to insufficient coverage. The Δ168 deletion was called correctly in both samples for which the deletion call was incorrect using the modified medaka pipeline. Nonetheless, it has to be mentioned that the longshot filter step with the medaka pipeline seems to help to reduce false-positive SNPs, which in case of the samples analyzed for this study occurred more frequent and in part systematically when using the nanopolish pipeline (data not shown).

## Faulty settings in some pipeline implementations for Illumina sequencing lead to hidden SNVs in primer-binding regions

When inspecting the consensus sequences and raw data of some of the seven of the ”escapee sequences”, it became apparent that a C>T transversion at position 14,599 was missing from the submitted consensus sequences. Position 14,599 is covered by primer nCoV-2019_48_RIGHT of the ARTIC-V3 amplicon scheme and faulty or disabled primer-trimming during data processing prior to SNP calling could hide the respective mutation. The COG-UK Illumina pipeline correctly called the mutation when reanalyzing the raw data. Nonetheless, this requires further attention, since about 5200 bases (17.7%) of the SARS-CoV-2 genome are covered with primer-binding sites when using the ARTIC-V3 amplicon scheme and obviously there are widely-used data analysis pipelines that do not correctly handle the respective positions.

## Coverage based sequencing depth normalization

The ARTIC pipeline includes the script align_trim.py that limits the sequencing depth of amplicons to a given limit N prior to analysis. This is performed by assigning each read to a single contig, based on the closest pair of amplicon primers in the primer pool, and counting the number of reads associated with each contig until a maximum of N reads per strand and amplicon were selected. All additional reads of an amplicon are discarded. Since we used 1200 bp amplicon libraries that were partly sequenced using ONT's Rapid Sequencing Kit RBK004 (as described in dx.doi.org/10.17504/protocols.io.bh7hj9j6), resulting in an uneven coverage distribution with lower coverage towards the ends of each amplicon, we adapted the sequencing depth normalization to a coverage-based instead of counter-based approach: The coverage of both strands is tracked in terms of start and end points of alignments. For each accepted read, the coverage in between the alignment start and alignment end position is increased for the reference strand to which it aligns. A read is dropped only if the strand-specific coverage of every position in the aligned region is already equal to or above the requested normalization threshold N. Effectively, this changes the behavior of the align_trim script such that it makes the normalization limit N a lower boundary instead of an upper boundary. A related pull request was created for the Github repository of the ARTIC pipeline (https://github.com/artic-network/fieldbioinformatics/pull/71). For the datasets in this study, we used a normalization limit of 200 instead of the default value of 100.
