# Supplementary figures and images for: Multiple Occurrences of a 168-Nucleotide Deletion in SARS-CoV-2 ORF8, Unnoticed by Standard Amplicon Sequencing and Variant Calling Pipelines
Source: Viruses. 2021 Sep 18;13(9):1870. doi: 10.3390/v13091870 (PMC8518987; doi:10.3390/v13091870)

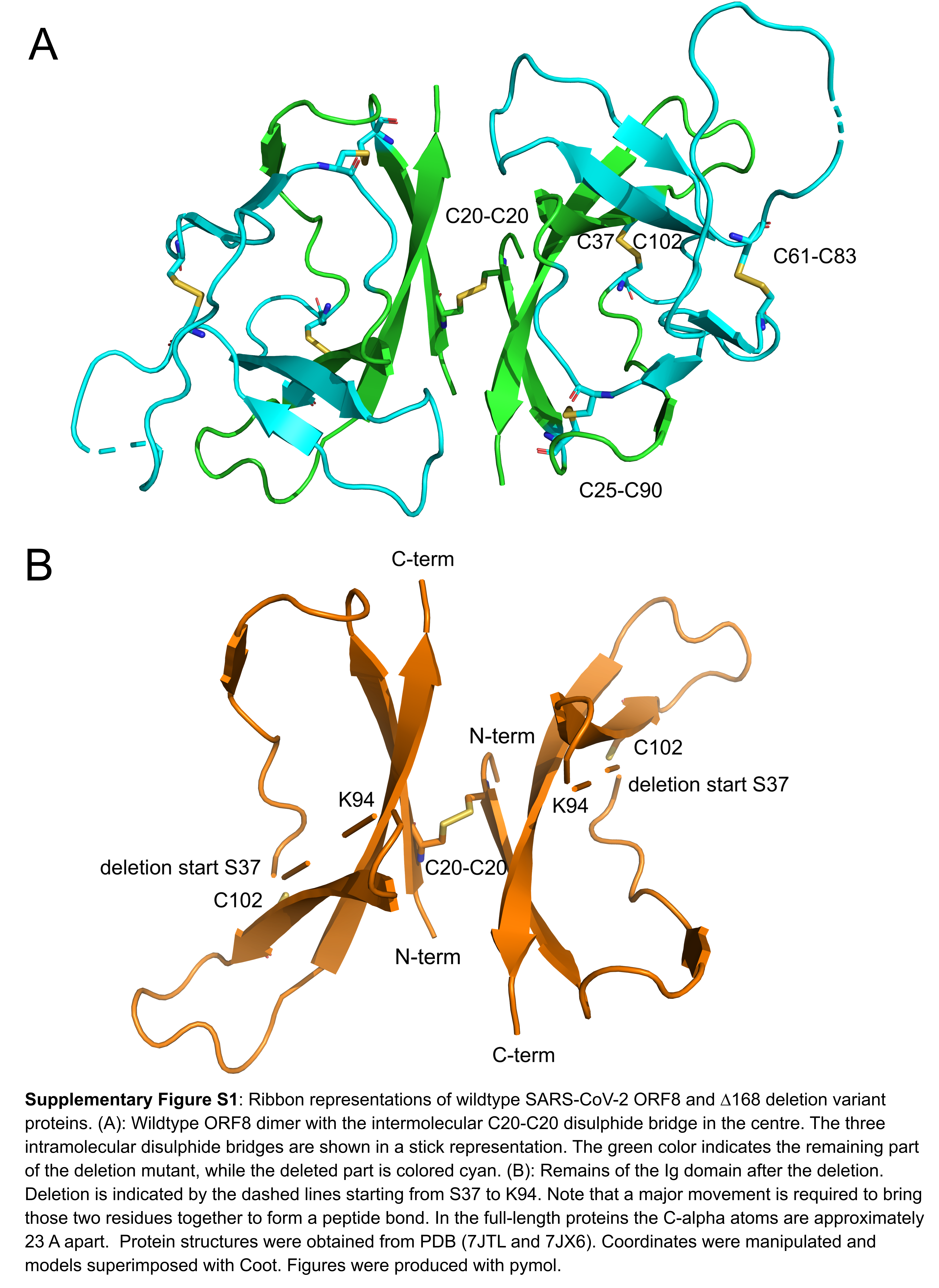

Supplement: Supplementary file 1 [file viruses-13-01870-s001.zip › Brandt_et_al._Viruses_Supplementary_Figure_S1_rev1.tiff]

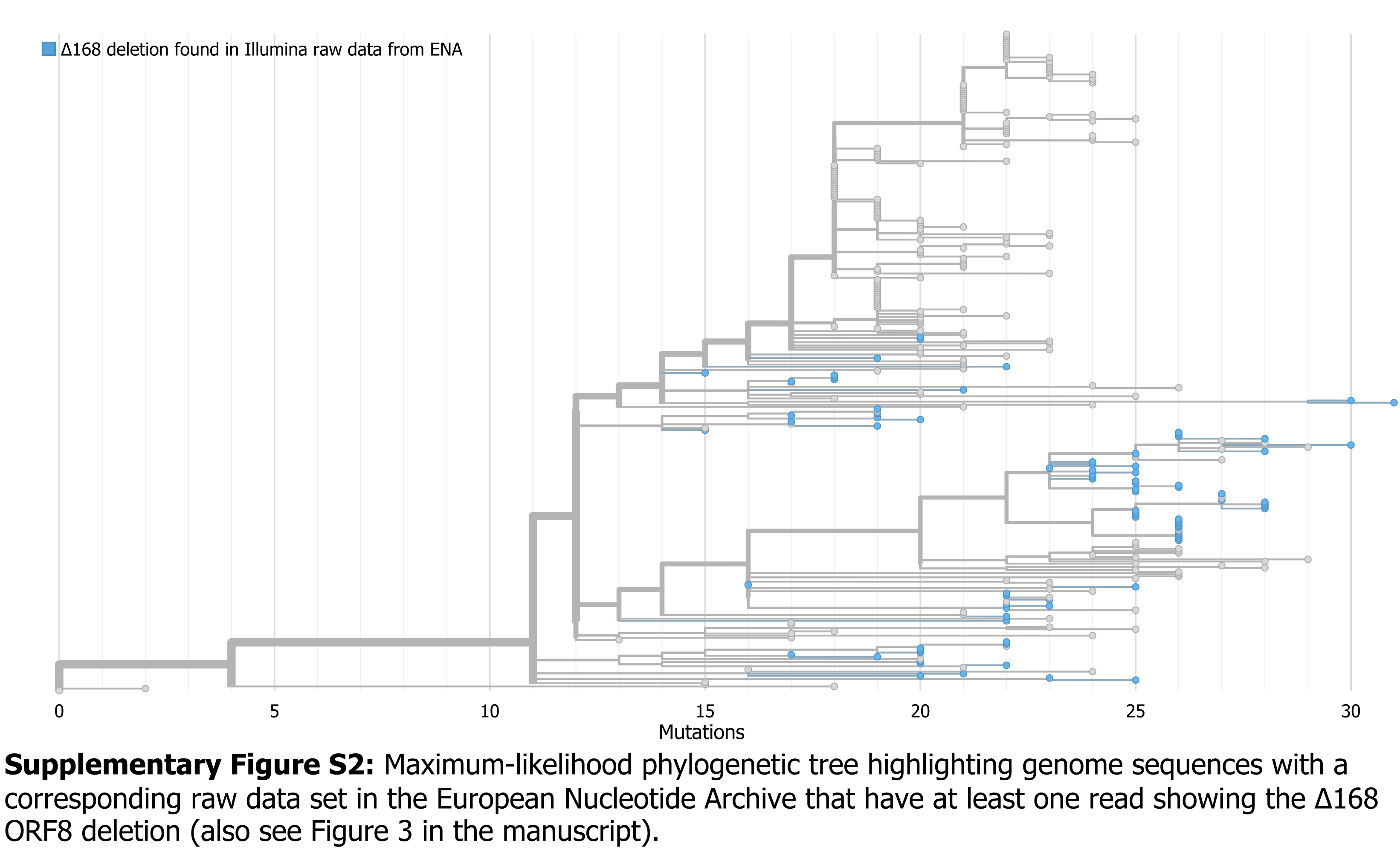

Supplement: Supplementary file 1 [file viruses-13-01870-s001.zip › Brandt_et_al._Viruses_Supplementary_Figure_S2_rev1.tiff]

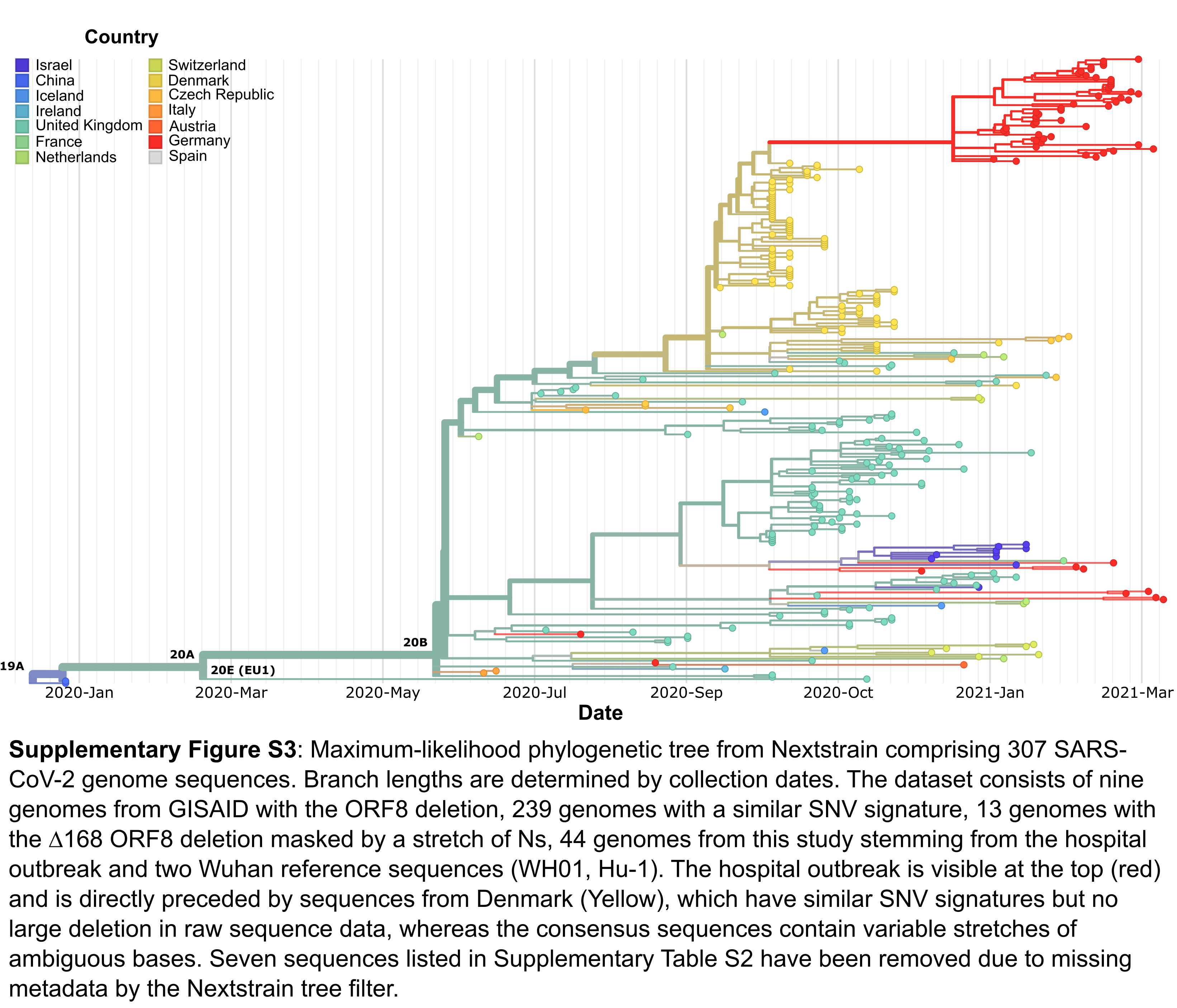

Supplement: Supplementary file 1 [file viruses-13-01870-s001.zip › Brandt_et_al._Viruses_Supplementary_Figure_S3_rev1.tiff]

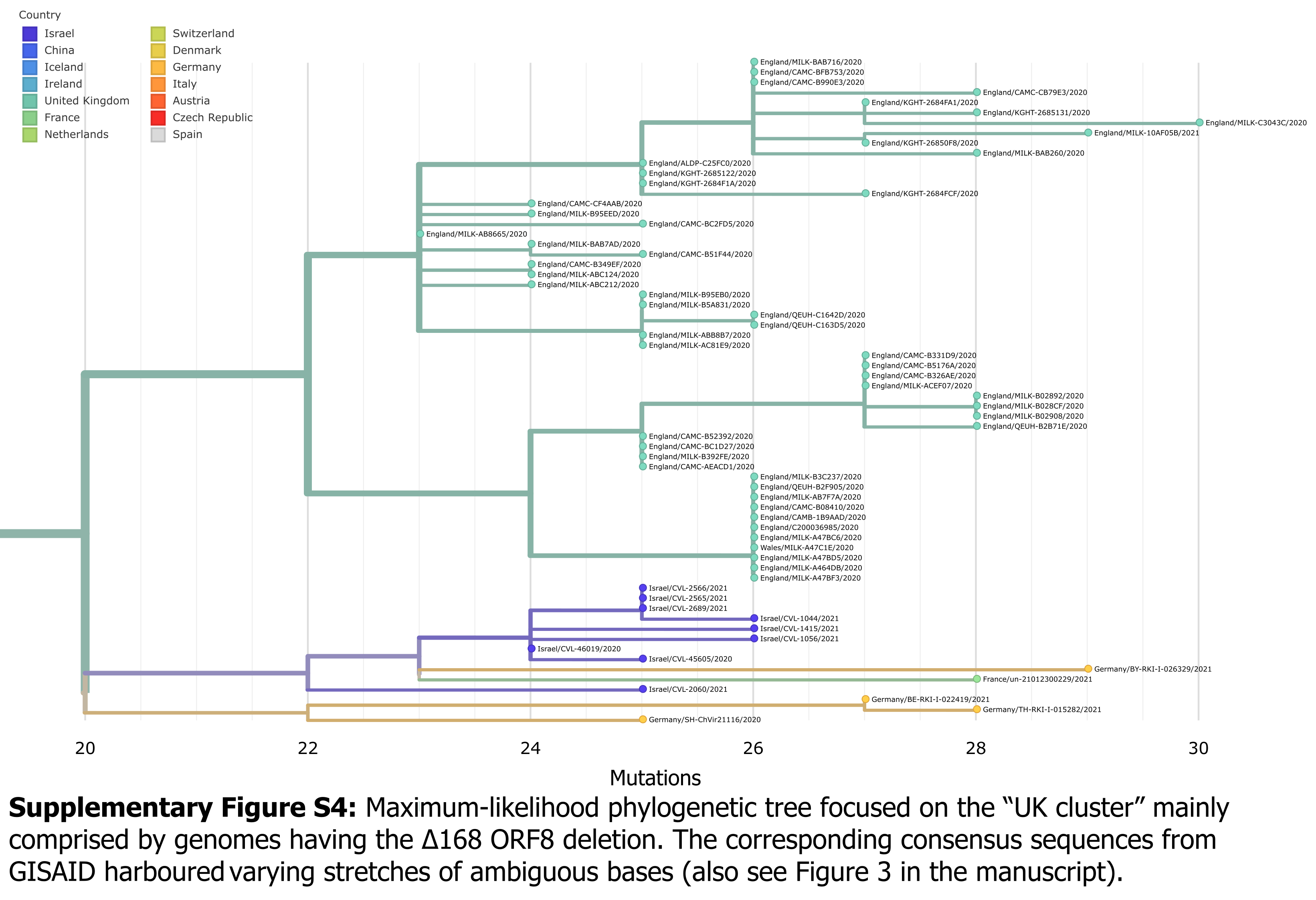

Supplement: Supplementary file 1 [file viruses-13-01870-s001.zip › Brandt_et_al._Viruses_Supplementary_Figure_S4_rev1.tiff]
